# Adaptive servo-ventilation therapy using an innovative ventilator for patients with chronic heart failure: a real-world, multicenter, retrospective, observational study (SAVIOR-R)

**DOI:** 10.1007/s00380-014-0558-8

**Published:** 2014-08-08

**Authors:** Shin-ichi Momomura, Yoshihiko Seino, Yasuki Kihara, Hitoshi Adachi, Yoshio Yasumura, Hiroyuki Yokoyama

**Affiliations:** 1 Cardiovascular Medicine, Saitama Medical Center, Jichi Medical University, 1-847 Amanuma-Cho, Omiya-Ku, Saitama, 330-8503 Japan; 2 Cardiovascular Center, Nippon Medical School Chiba Hokusoh Hospital, Chiba, Japan; 3 Department of Cardiovascular Medicine, Hiroshima University, Hiroshima, Japan; 4 Division of Cardiology, Gunma Prefectural Cardiovascular Center, Gunma, Japan; 5 Cardiovascular Division, Osaka National Hospital, Osaka, Japan; 6 Department of Cardiovascular Medicine, National Cerebral and Cardiovascular Center, Osaka, Japan

**Keywords:** Adaptive servo-ventilation, Chronic heart failure, Noninvasive positive pressure ventilation, Cardiac function, Sleep-disordered breathing

## Abstract

Adaptive servo-ventilation (ASV) therapy using an innovative ventilator—originally developed to treat sleep-disordered breathing (SDB)—is a novel modality of noninvasive positive pressure ventilation and is gaining acceptance among Japanese cardiologists in expectation of its applicability to treat patients with chronic heart failure (CHF) based on its acute beneficial hemodynamic effects. We conducted a multicenter, retrospective, real-world observational study in 115 Japanese patients with CHF, who had undergone home ASV therapy for the first time from January through December 2009, to examine their profile and the effects on their symptoms and hemodynamics. Medical records were used to investigate New York Heart Association (NYHA) class, echocardiographic parameters including left ventricular ejection fraction (LVEF), cardiothoracic ratio (CTR), brain natriuretic peptide (BNP), and other variables. Most of the patients were categorized to NYHA classes II (44.4 %) and III (40.7 %). SDB severity was not determined in 44 patients, and SDB was not detected or was mild in 27 patients. In at least 71 patients (61.7 %), therefore, ASV therapy was not applied for the treatment of SDB. CHF was more severe, i.e., greater NYHA class, lower LVEF, and higher CTR, in 87 ASV-continued patients (75.7 %) than in 28 ASV-discontinued patients (24.3 %). However, SDB severity was not related to continuity of ASV. The combined proportion of NYHA classes III and IV (*P* = 0.012) and LVEF (*P* = 0.009) improved significantly after ASV therapy. CTR and BNP did not improve significantly after ASV therapy but showed significant beneficial changes in their time-course analysis (*P* < 0.05, respectively). Improvements in LVEF and NYHA class after ASV therapy were not influenced by SDB severity at onset. The present study suggests that ASV therapy would improve the symptoms and hemodynamics of CHF patients, regardless of SDB severity. A randomized clinical study to verify these effects is warranted.

## Introduction

Chronic heart failure (CHF) is the end-stage pathology of all heart diseases [1], and pharmacotherapy is the first-line therapy for patients with CHF. Patient prognosis was considerably improved by the diffusion of pharmacotherapy and by the recent striking progress in non-pharmacotherapy including cardiac resynchronization therapy (CRT) [2–6]. Nevertheless, CHF is a leading cause of cardiovascular death [7, 8], and patients with CHF repeat admissions for acute exacerbation of CHF.

Noninvasive positive pressure ventilation (NPPV) has been shown to improve pulmonary congestion of patients with acute heart failure (AHF) and in the acute exacerbation of CHF through the following hemodynamic actions: re-opening of collapsed alveoli, prevention of small airway obstruction, enlargement of lung volume, improvements in oxygenation and lung compliance [9–15], amelioration of left ventricular afterload through a reduction in transmural pressure induced by positive intrathoracic pressure [13, 14], and relief of left ventricular preload through a reduction in venous return [10, 15]. Based on these acute beneficial effects of NPPV, cardiologists had been aware of the potential applicability of NPPV to the treatment of CHF patients. However, such application was very difficult to realize because conventional ventilators used for NPPV presented poor tolerability and cumbersome operability. Therefore, the development of an innovative ventilator capable of solving these drawbacks was expected. The ventilator used for adaptive servo-ventilation (ASV), a form of NPPV, offers superior tolerability and simple operability based on the provision of support pressure; the device was originally developed to treat sleep-disordered breathing (SDB) [16] and is synchronized to the respiration patterns of individual patients through its original algorithm and potentially allows for the application of home ASV therapy to the treatment of CHF patients.

In recent years, ASV therapy diffused rapidly and widely in Japan and is gaining acceptance among cardiologists. A number of clinical studies [17–21] on ASV therapy have been published. However, no clinical evidence of ASV therapy in real-world patients at multiple medical institutions has been available to date. The objectives of the present study were to investigate the actual practice of ASV therapy for patients with CHF in Japan and to examine the effects of ASV therapy on their symptoms and hemodynamics.

## Patients and methods

### Patients

Among Japanese outpatients with CHF who had been treated at 16 medical institutions, 116 patients were enrolled (1) who for the first time had undergone home ASV therapy from January through December 2009, (2) who aged 20 years or older at the onset of ASV therapy, and (3) who did not fall under the exclusion criterion (patients considered by their attending physician to be ineligible for the present study). Furthermore, one of them was excluded because of discovering the non-outpatient nature of the patient after enrolment. In consequence, 115 patients (90 males and 25 females) were analyzed for the efficacy of ASV therapy. The present study was conducted after the acquisition of approval by the ethics committee at each participating institution and in accordance with the Declaration of Helsinki.

### Study design and method

The present study is a multicenter retrospective observational study in Japanese patients with CHF in real-world settings. In principle, medical records prepared for 1 year before and after the onset of ASV therapy were used to investigate the following items: regarding patient background, age, gender, underlying heart disease, complications, cardiovascular events, and others; regarding efficacy, vital signs, symptoms of CHF, New York Heart Association (NYHA) functional class, hematology, human brain natriuretic peptide (BNP), echocardiography determining left ventricular ejection fraction (LVEF), left ventricular end-systolic dimension (LVDs), left ventricular end-diastolic dimension (LVDd), and left atrial dimension (LAD), chest X-ray documenting the cardiothoracic ratio (CTR), sleep study, estimated glomerular filtration rate (eGFR), and others; and regarding continuity, ventilator use and others. Examinations were performed in accordance with the standards valid at each participating institution. The present study did not assess the safety of ASV therapy.

We used the following two categories of definitions for the “baseline values”: (1) for the statistical analysis of the pre- and post-ASV values, “the values that were obtained closest to the onset of ASV therapy in a range from day −363 to day 7”; and (2) for the statistical analysis of the time-course changes in variables, “the values that were obtained closest to the onset of ASV in a range from day −56 to day 14.” Furthermore, we used the following definition for the post-ASV values: “the values that were obtained latest since day 8 after the onset of ASV therapy.” In addition, we established the allowable ranges of ± 28 days for each of the other assessment points.

### Ventilator used for ASV therapy

The ventilator for ASV therapy used in the present study was an advanced bilevel positive airway pressure unit—AutoSet™ CS (ResMed, Sydney, Australia). The device learns the patient’s breathing rates and patterns, provides proper pressure support that is synchronized to them through its state-of-the-art fuzzy logic algorithms, and generates smooth pressure waveforms mimicking the patient’s normal respiration flow patterns. ASV therapy at home was conducted in patients whose symptoms were stable and for whom the attending physician considered it appropriate. The device is used confinedly in the range of coverage by the National Health Insurance System in Japan. The application of ASV therapy to the treatment of SDB is currently not covered by the system.

### Statistical analyses

The statistical analyses to compare the pre- and post-ASV values were performed using paired *t* test, one-sample Wilcoxon’s signed rank sum test, and McNemar’s test for parametric, nonparametric, and binary variables, respectively. Subgroup analyses were performed using the generalized estimating equation procedure to examine time-course changes in continuous and categorical variables, followed by the least Fisher’s significant difference method to determine the timing for generation of a statistically significance difference. Stratified analyses to identify the background factors impacting on the continuity and efficacy of ASV therapy were conducted using Student’s *t* test, two-sample Wilcoxon’s signed rank sum test, and Fisher’s exact probability test for parametric, nonparametric, and binary variables, respectively. Furthermore, multivariate logistic regression analysis using Wald *χ* ^2^ test was performed to identify patients’ background factors associated with LVEF improvement. A value of *P* < 0.05 was considered statistically significant. All statistical analyses were performed using a statistical software package, version 9.2 (SAS Institute Inc., Cary, NC, USA).

## Results

### Characteristics of CHF patients who underwent ASV therapy

Characteristics of 115 patients at the onset of ASV therapy are shown in Table 1. Mean age was 64.7 ± 12.7 years, male gender was predominant—78.3 %, the proportion of patients with dilated cardiomyopathy (DCM) was 37.4 %, and patients having a disease duration of ≥1 year accounted for 67.8 %. At onset, more than 80 % of patients received diuretics and beta blockers, and approximately 80 % of patients were medicated with angiotensin-converting enzyme inhibitors and angiotensin II receptor blockers. Despite the fact that patients had already undergone the sufficient treatment of their heart failure (HF), the combined proportion of patients with NYHA class III and IV HF was as high as 43.2 %, mean LVEF was 37.9 %, mean CTR was 56.7 %, and median plasma BNP concentration was 312.8 pg/mL. Therefore, the majority of patients on ASV therapy were found to have severe CHF. All patients underwent ASV therapy providing end-expiratory pressure, minimum pressure support, and maximum pressure support. The default values, the number of patients who used the device under default settings, and the range for these pressures were, respectively, as follows: 5, 3, and 10; 81, 112, and 105; and 3–8, 3–4, and 8–12 cmH_2_O.

**Table 1:**
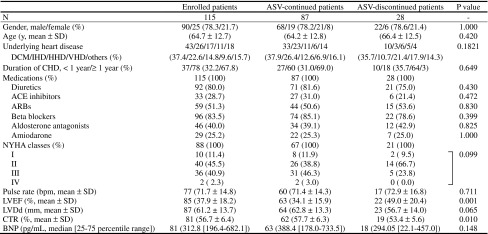
Baseline characteristics of enrolled patients, ASV-continued patients, and ASV-discontinued patients

*ASV* adaptive servo-ventilation, *DCM* dilated cardiomyopathy, *IHD* ischemic heart disease, *HHD* hypertensive heart disease, *VHD* valvular heart disease, *CHF* chronic heart failure, *ACE* angiotensin-converting enzyme, *ARBs* angiotensin receptor blockers, *NYHA* New York Heart Association, *bpm* beats per minute, *LVEF* left ventricular ejection fraction, *LVDd* left ventricular end-diastolic dimension, *CTR* cardiothoracic ratio, *BNP* brain natriuretic peptide

The *P* values were calculated between ASV-continued and -discontinued patients according to Student’s *t* test, two-sample Wilcoxon’s signed rank sum test, or Fisher’s exact probability test

### Patient disposition

**Fig. 1:**
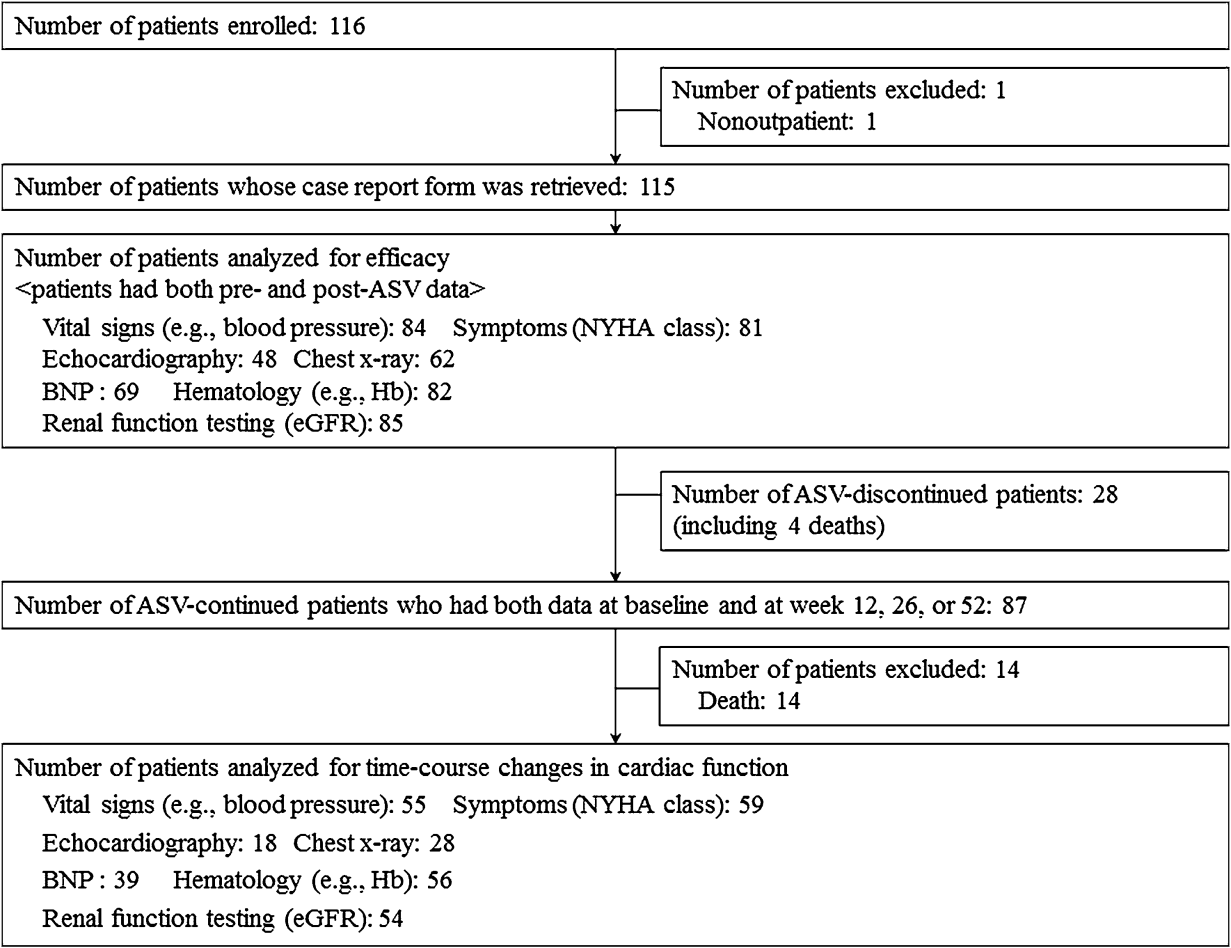
Patient disposition. *ASV* adaptive servo-ventilation, *NYHA* New York Heart Association, *BNP* brain natriuretic peptide, *Hb* hemoglobin, *eGFR* estimated glomerular filtration rate

Patient disposition is shown in Fig. 1. The retrieval rate of the case report form on 115 patients who were analyzed for efficacy was 100 %; the attending physician had discontinued ASV therapy at his/her discretion in 28 patients (24.3 %) of them (ASV-discontinued patients). The most predominant reason for discontinuation of ASV therapy was “impatience” (20 patients, 71.4 %), followed by “economic reason” (4 patients, 14.3 %), “improvement in HF” (2 patients, 7.1 %), and “deterioration of HF” (2 patients, 7.1 %). In contrast, the attending physician had continued ASV therapy at his/her discretion in 87 patients (75.6 %) of them (ASV-continued patients), 13 of whom died due to the progression of HF. The proportions of patients were 40.9 % (47/115) to 73.9 % (85/115), who were analyzed for seven investigation items: vital signs [body weight, pulse rate, systolic blood pressure (SBP), and diastolic blood pressure (DBP)], echocardiography, BNP, renal function test, symptoms of CHF, chest X-ray, and hematology.

Among 115 patients who were analyzed for efficacy, 18 died within 1 year after the onset of ASV therapy: 16 died due to the spontaneous deterioration of HF or to lethal arrhythmias, 1 to suicide, and 1 to ileus. It was the attending physician who had found no causality between ASV therapy and death at his/her discretion.

### Sleep study at the onset of ASV therapy

**Fig. 2:**
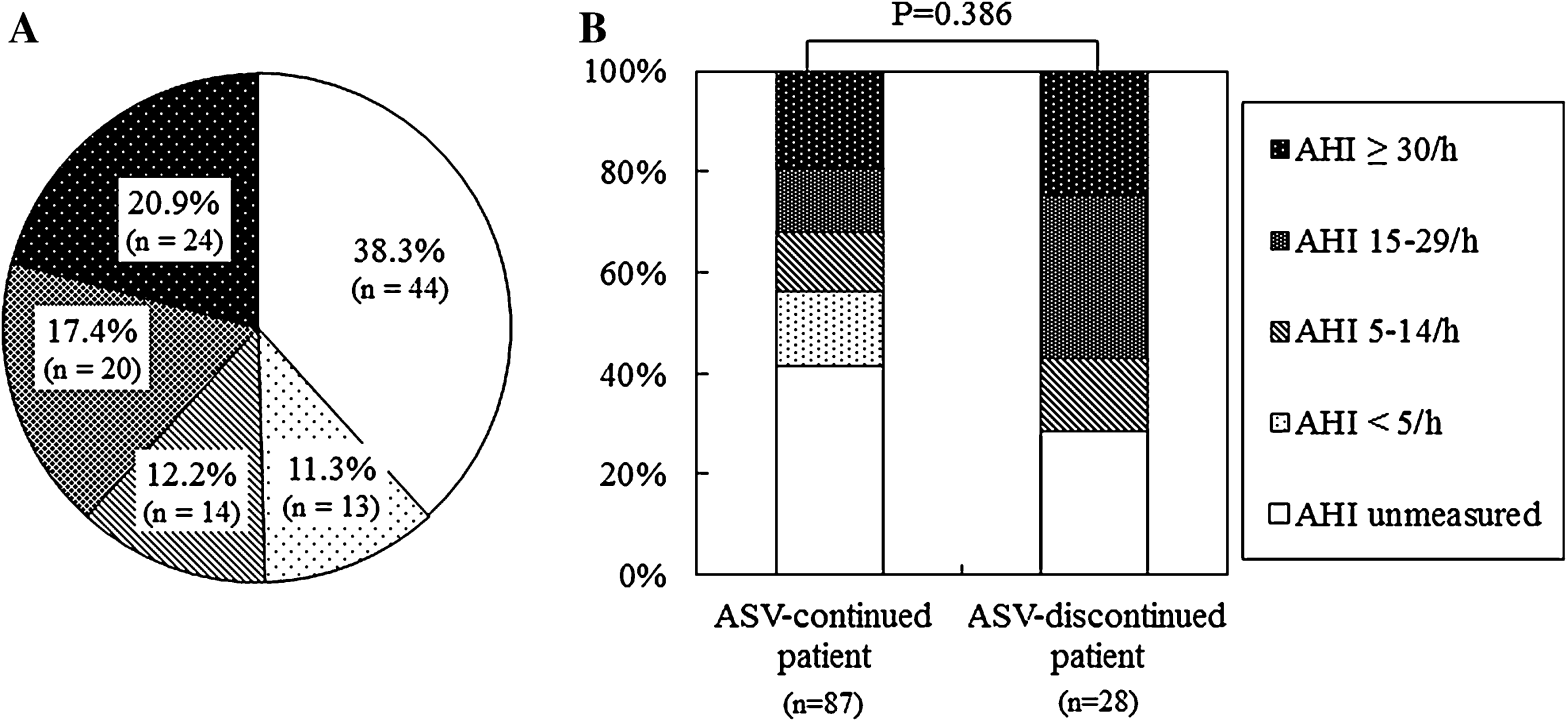
**a** Diagram showing the results of AHI measurements at onset in CHF patients who underwent ASV therapy using an innovative ventilator. **b** Results from the stratified analysis on AHI distributions in the subgroups of ASV-continued and -discontinued patients. The *P* value was calculated between ASV-continued and -discontinued patients according to Wilcoxon’s rank sum test. *ASV* adaptive servo-ventilation, *AHI* apnea–hypopnea index

The status of conducting the sleep study at the onset of ASV therapy is shown in Fig. 2a. Patients, who underwent the study, were assessed for the severity of SDB by means of the apnea–hypopnea index (AHI). Consequently, SDB was present in 50.4 % (58/115) of patients. The percentages of patients with mild, moderate, and severe SDB were 12.2 % (14/115), 17.4 % (20/115), and 20.9 % (24/115), respectively. Patients with CHF who were complicated by moderate or severe SDB accounted for 38.3 % (44/115) of patients. On the other hand, the proportions of patients whose SDB severity was not assessed because the sleep study was not performed and of patients who were not complicated by SDB were 38.3 % (44/115) and 11.3 % (13/115), respectively. Namely, ASV therapy was not applied for the objective of treating SDB in at least 61.7 % of patients.

### Subgroup analyses of variables for SDB, HF, and hemodynamics between ASV-discontinued patients and ASV-continued patients

The results from the stratified analysis on AHI distributions in the subgroups of ASV-continued and -discontinued patients are shown in Fig. 2b. The mean pre-ASV values of AHI were 24.0 ± 21.3 and 28.8 ± 19.2/h in ASV-continued patients and ASV-discontinued patients, respectively, with no statistically significant difference (*P* = 0.386). However, the combined proportions of NYHA class III and IV HF patients (*P* = 0.047), LVEF (*P* = 0.001), and CTR (*P* = 0.010) at the onset of ASV therapy were significantly higher, lower, and greater, respectively, in ASV-continued patients than in ASV-discontinued patients (Table 1).

### NYHA classification

**Fig. 3:**
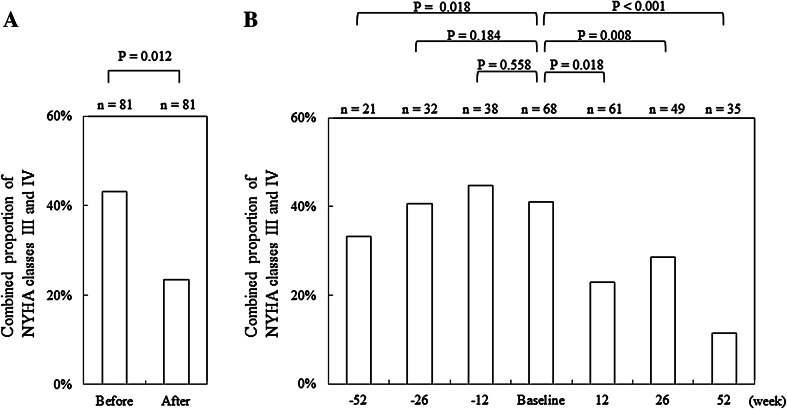
**a** Diagram showing the combined proportions of NYHA classes III and IV before and after ASV therapy. The *P* values were calculated according to McNemar’s test. **b** Diagram showing time-course changes in the combined proportions of NYHA classes III and IV by assessment week. The *P* values were calculated according to Fisher’s least significant difference method. *NYHA* New York Heart Association, *ASV* adaptive servo-ventilation

Figure 3a indicates pre- and post-ASV therapy changes in the combined proportion of patients with NYHA class III and IV. Time-course changes before and after ASV therapy in the proportion are shown in Fig. 3b. Eighty-one patients showed no significant difference in the proportion during 1 year before the onset of ASV therapy. After ASV therapy, however, the proportion significantly decreased (*P* = 0.012) from the pre-ASV value of 43.2 % to the post-ASV value of 23.4 %. Figure 3b indicates time-course changes by treatment week in the proportion. The statistical analysis of the proportions between weeks −52, −26, and −12 and onset revealed a significant increase (*P* = 0.018) from 33.3 to 41.2 % only between week −52 and onset. In contrast, the statistical analysis of the proportions between onset and weeks 12, 26, and 52 disclosed significant decreases (*P* = 0.018, *P* = 0.008, and *P* < 0.001) against the onset value at week 12 and later. Thus, the distribution patterns of NYHA classes also favorably changed after ASV therapy (Table 2; *P* = 0.001) and at weeks 26 and 52 of treatment (Table 3; *P* = 0.008 and *P* < 0.001, respectively). The abovementioned improvements in NYHA class were highly likely to be attributable to ASV therapy because patients showing improvements in NYHA class had a significantly greater number (*P* < 0.001) of days of ASV therapy (frequency × duration).

**Table 2:**
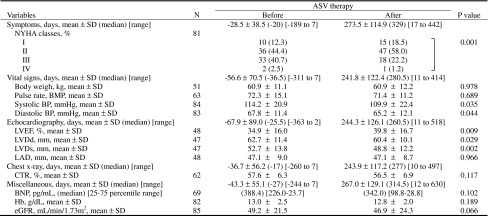
Variables before and after ASV therapy

*ASV* adaptive servo-ventilation, *NYHA* New York Heart Association, *BPM* beats per minute, *BP* blood pressure, *LVEF* left ventricular ejection fraction, *LVDd* left ventricular end-diastolic dimension, *LVDs* left ventricular end-systolic dimension, *LAD* left atrial dimension, *CTR* cardiothoracic ratio, *BNP* brain natriuretic peptide, *Hb* hemoglobin, *eGFR* estimated glomerular filtration rate

The *P* values were calculated according to paired *t* test or one-sample Wilcoxon’s signed rank sum test

**Table 3:**
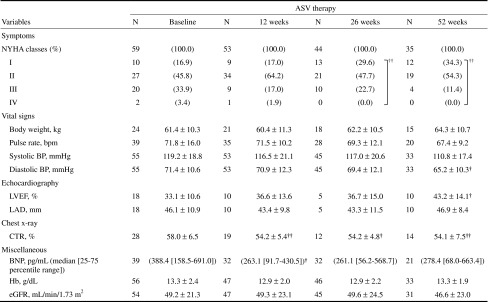
Variables at baseline and ASV therapy weeks

Values are expressed as mean ± SD

*ASV* adaptive servo-ventilation, *NYHA* New York Heart Association, *bpm* beats per minute, *BP* blood pressure, *LVEF* left ventricular ejection fraction, *LAD* left atrial dimension, *CTR* cardiothoracic ratio, *BNP* brain natriuretic peptide, *Hb* hemoglobin, *eGFR* estimated glomerular filtration rate Values are expressed as mean ± SD. Significant difference versus baseline (Fisher’s least significant difference method, *P* < 0.05 or *P* < 0.01, respectively)

^†, ††^Significant difference versus baseline (Fisher’s least significant difference method, *P* < 0.05 or *P* < 0.01, respectively)

### Echocardiography

**Fig. 4:**
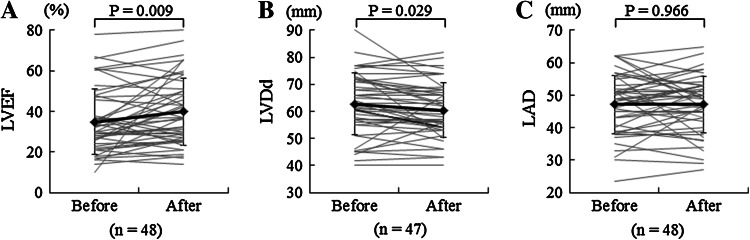
Changes in cardiac function and dimensions by echocardiography after ASV therapy. The *P* values were calculated according to paired *t* test. *ASV* adaptive servo-ventilation, *horizontal lines* data of individual patients, *bold horizontal lines* means, *I bars* ± SD, *LVEF* left ventricular ejection fraction, *LVDd* left ventricular end-diastolic dimension, *LAD* left atrial dimension

The mean pre- and post-ASV values of LVDd and LVDs were 62.7 and 60.4 mm, as well as 52.7 and 48.8 mm, respectively; therefore, both variables decreased significantly (*P* = 0.029 and *P* = 0.002, respectively). In association with these changes, LVEF increased significantly (*P* = 0.009) from the mean pre-ASV value of 34.9 % to the mean post-ASV value of 39.8 % (Fig. 4; Table 2). Furthermore, statistical analysis by treatment week revealed that LVEF tended to increase at weeks 12 and 26 of treatment and increased significantly (*P* = 0.035) at week 52 of treatment. LAD remained unchanged after ASV and between baseline and each treatment week (Table 3).

### Vital signs

Body weight and pulse rate changed neither in the comparison of the pre- and post-ASV values nor in statistical analysis by treatment week. After ASV therapy, SBP and DBP decreased significantly against the pre-ASV values (*P* = 0.035 and *P* = 0.044, respectively). However, SBP showed no significant change in statistical analysis by treatment week. Furthermore, DBP showed a significant decrease (*P* = 0.043) against the onset value only at week 52 of treatment (Tables 2, 3).

### Other examinations

After ASV therapy, BNP, Hb, eGFR, and CTR showed no statistically significant difference against the pre-ASV values. However, statistical analysis by treatment week revealed a significant decrease (*P* = 0.017) in BNP only at week 12 of treatment from the median onset value of 388.4 pg/mL to the median post-ASV value of 263.1 pg/mL, as well as significant decreases in CTR at weeks 12, 26, and 52 of treatment from the onset values (58.0 %), 54.2, 54.2 and 54.1 %, respectively (*P* = 0.003, *P* = 0.019, and *P* = 0.010, respectively) (Tables 2, 3).

### Stratified analyses of the combined proportions of NYHA class III and IV HF patients and of LVEF

**Fig. 5:**
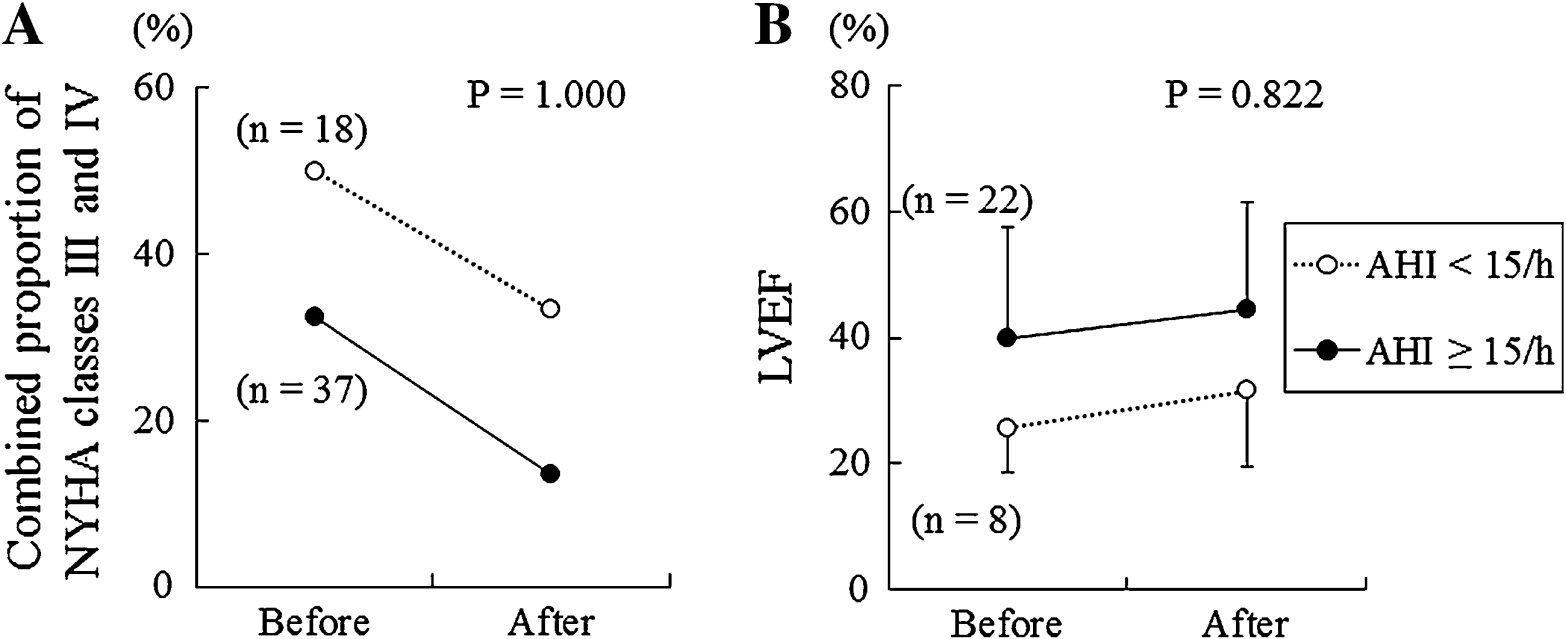
Stratified analysis in the combined proportion of NYHA classes III and IV and LVEF before and after ASV therapy, with an AHI cutoff value of 15/h. **a** Diagram showing changes in AHI in relation to the proportion of NYHA classes III and IV after ASV therapy. **b** Diagram showing changes in AHI in relation to LVEF after ASV therapy. Values are expressed as mean ± SD. The *P* values were calculated according to Fisher’s exact probability test or Student’s *t* test. *ASV* adaptive servo-ventilation, *AHI* apnea–hypopnea index, *NYHA* New York Heart Association, *LVEF* left ventricular ejection fraction

The results of the statistical analyses on the combined proportions of NYHA class III and IV HF patients and LVEF, which were found after ASV therapy using the AHI value of 15/h as the cutoff value, are shown in Fig. 5. Between the subgroup of patients with AHI (≥15/h) and the subgroup of patients with AHI (<15/h), no significant difference was found in the combined proportion of NYHA class III and IV HF patients (Fig. 5a) or LVEF (Fig. 5b). However, patients with a pre-ASV value of AHI (<15/h) tended to have severe HF as compared to patients with a pre-ASV value of AHI (≥15/h). This finding is not concordant with previous clinical studies that have recognized an association between SDB severity and HF severity. We speculate that this discordance is attributable to the fact that in Japan ASV therapy is conducted in expectation of SDB improvement and CHF patient’s hemodynamic improvement. Furthermore, SDB severity was not related to continuity of ASV.

### Multivariate logistic regression analysis

The results of the multivariate logistic regression analysis on independent variables associated with LVEF improvement (any numerical increase from the pre-ASV value), with and without AHI as a background factor, are shown in Tables 4 and 5, respectively.

**Table 4:** Logistic regression analysis of patients’ background factors associated with LVEF improvement when not including AHI

Background factors	Likelihood ratios					Odds ratios	
	*B*	SE	Wald *χ* ^2 a^	*df*	*P* value^b^	Exp(*B*)	95 % Wald CI
Age (≥ 65 years)	1.850	0.907	4.163	1	0.041	6.362	(1.076–37.632)
CKD	−2.842	1.011	7.904	1	0.005	0.058	(0.008–0.423)
LVEF at baseline (< 40 %)	−0.066	0.027	5.741	1	0.017	0.936	(0.887–0.988)

*B* coefficient for the logistic regression equation to predict the dependent variable from the independent variable, *SE* standard error around the coefficient, *df* degree of freedom for Wald *χ* ^2^ test, *Exp*(*B*) exponentiation of the *B* coefficient, an odds ratio, *LVEF* left ventricular ejection fraction, *AHI* apnea–hypopnea index, *CI* confidence interval, *CKD* chronic kidney disease

^a^Wald *χ* ^2^ statistic

^b^A value of *P* < 0.05 was considered statistically significant

**Table 5:** Logistic regression analysis of patients’ background factors associated with LVEF improvement when including AHI

Background factors	Likelihood ratios					Odds ratios	
	*B*	SE	Wald *χ* ^2 a^	*df*	*P* value^b^	Exp(*B*)	95 % Wald CI
Age (≥ 65 years)	2.929	1.761	2.768	1	0.096	18.711	(0.594–589.686)
CKD	−4.187	1.862	5.059	1	0.025	0.015	(< 0.001–0.584)
LVEF at baseline (< 40 %)	−0.077	0.044	3.094	1	0.079	0.926	(0.850–1.009)
AHI at baseline (≥ 15/h)	−0.887	1.575	0.317	1	0.573	0.412	(0.019–9.020)

*B* coefficient for the logistic regression equation to predict the dependent variable from the independent variable, *SE* standard error around the coefficient, *df* degree of freedom for Wald *χ* ^2^ test, *Exp*(*B*) exponentiation of the *B* coefficient, an odds ratio, *LVEF* left ventricular ejection fraction, *AHI* apnea–hypopnea index, *CI* confidence interval, *CKD* chronic kidney disease

^a^Wald *χ* ^2^ statistic

^b^A value of *P* < 0.05 was considered statistically significant

The backward selection method was used to analyze background factors [gender, age (≥65 years), underlying heart disease (DCM, ischemic heart disease, hypertensive heart disease, valvular heart disease, and others), diabetes mellitus, hypertension, chronic kidney disease (CKD), atrial fibrillation, medications [e.g., diuretics, beta blockers, and angiotensin receptor blockers (ARBs)], and LVEF at onset], and variables to perform the multivariate logistic regression analysis were selected. Consequently, age (≥65 years), CKD, and LVEF at onset (≤40 %) were selected as independent variables for the dependent variable—LVEF improvement. The Wald *χ* ^2^ value was significant (*P* < 0.05). Age showed a positive correlation [regression coefficient 1.850; odds ratio 6.362; 95 % confidence interval (CI) 1.076–37.632; *P* = 0.041] with LVEF improvement, while both CKD and LVEF at onset showed negative correlations (regression coefficient −2.842; odds ratio 0.058; 95 % CI 0.008–0.423; *P* = 0.005; and regression coefficient −0.066; odds ratio 0.936; 95 % CI 0.887–0.988; *P* = 0.017) (Table 4).

To determine the presence or absence of any contribution of AHI to the dependent variable, furthermore, the multivariate logistic regression analysis including AHI as an additional background factor was conducted. Consequently, AHI was found not to have any contribution to the dependent variable although the number of analyzable patients reduced to 28 from 48 (regression coefficient −0.887; odds ratio 0.412; 95 % CI 0.019–9.020; *P* = 0.573) (Table 5).

## Discussion

The present research is the first clinical study to investigate the actual practice of ASV therapy in Japan and enrolled 116 patients with CHF. There was only one exclusion as mentioned previously, indicating an exclusion rate as very low as 0.86 % (1/116). Furthermore, the retrieval rate of the case report form on the remaining 115 patients was 100 %. Therefore, the present study may be considered highly reliable as a field survey.

The ventilator, which had originally been developed to treat patients with SDB, was used despite the facts that 38.3 % of patients had no diagnosis of SDB and that 23.5 % of patients did not have or had mild SDB. Namely, we found that ASV therapy was conducted not for the treatment of SDB but for the improvement in hemodynamics in at least 61.7 % of patients with CHF at 16 medical institutions. This leads us to conjecture that ASV therapy, in practice, is presumably and widely applied to a much greater number of patients with impaired hemodynamics in whole Japan, regardless of the presence or absence of SDB. Furthermore, we found that ASV therapy represents a noninvasive therapeutic option for patients with intractable and relatively severe CHF in real-world settings because, at the onset of ASV therapy, patients with IHD accounted for approximately 20 %. The combined proportion of patients with NYHA classes III and IV HF was 43.2 % despite the high prescription rates of all drugs for the treatment of HF, and the median plasma BNP concentration was as high as 388.4 pg/mL.

The proportion of ASV-continued patients in the present study, 75.7 % (87/115), was higher than approximately 40–50 %—the values reported in previous nonrandomized studies [17, 22, 23]. This fact probably indicates good tolerance as the result that most of patients in the present study who had relatively severe CHF felt better comfort or became aware of improvements in their symptoms during ASV therapy, and is in line with a prior clinical study which suggested that compliance is a consequence of subjective benefits that patients experienced in their treatment [24]. Better comfort that patients obtained might have contributed to an improvement in their adherence to ASV therapy, and we consider that this good ASV therapy tolerance of CHF patients is translated into favorable NYHA class changes.

**Fig. 6:**
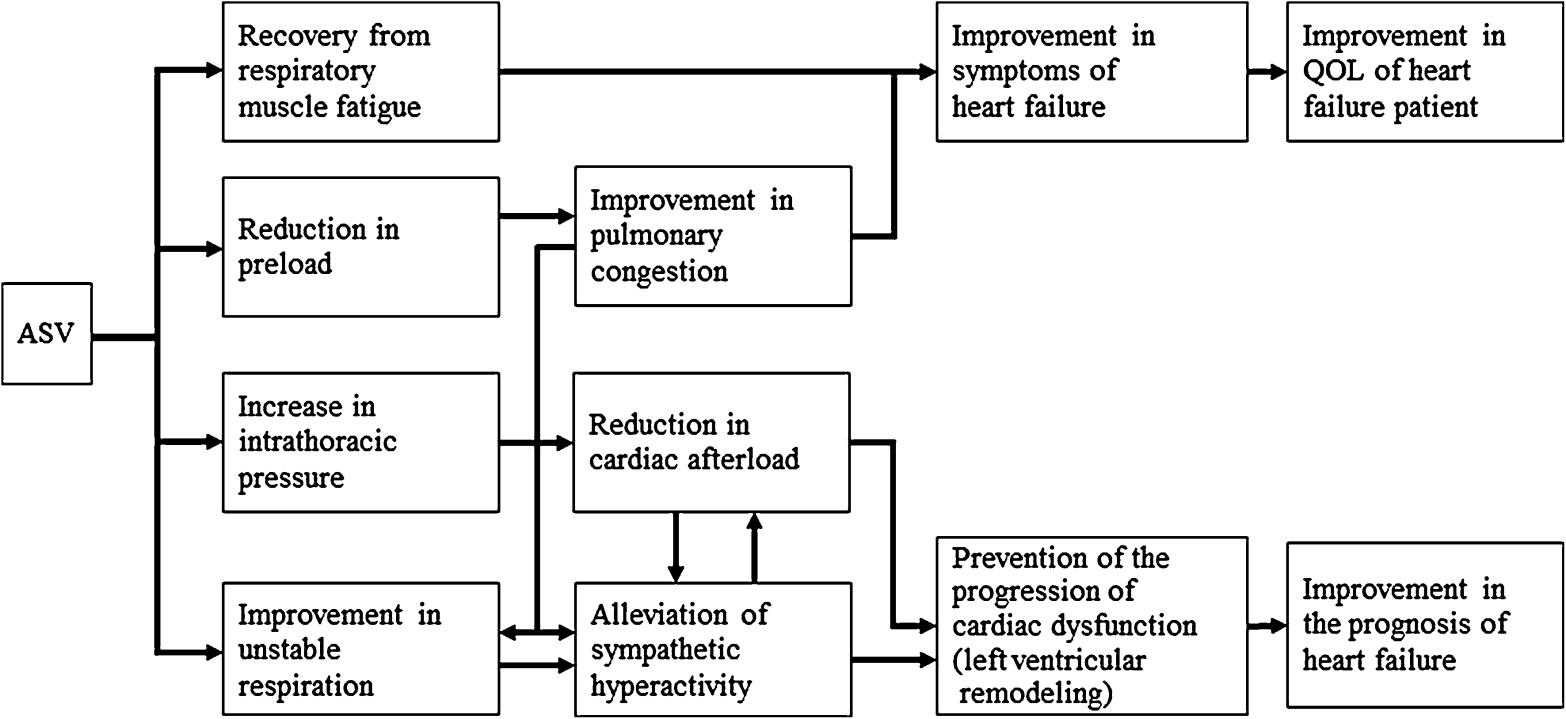
Postulated mechanisms by which ASV therapy improves the symptoms and hemodynamics of patients with CHF. *ASV* adaptive servo-ventilation, *CHF* chronic heart failure, *QOL* quality of life

Figure 6 illustrates the postulated mechanisms by which ASV therapy exerts its efficacy through improvements in the symptoms and hemodynamics of patients with CHF. ASV therapy using positive end-expiratory pressure (PEEP) alleviates preload through a reduction in venous return, which improves pulmonary congestion [10, 15]. PEEP ameliorates afterload by reducing transmural pressure through positive intrathoracic pressure, and ASV therapy—because of pressure support ventilation—unloads respiration muscles [13, 14]. Furthermore, an improvement in pulmonary congestion itself achieved by PEEP of ASV therapy probably inhibits sympathetic nerve activity because ASV therapy suppresses sympathetic nerve overactivity by decreasing pulmonary capillary wedge pressure through a reduction in venous return in CHF patients [25–27]. Via the mechanisms described above, ASV therapy is considered to improve symptoms of HF, to cause cardiac reverse remodeling [17–19], and eventually to achieve the therapeutic goals of CHF—improvements in the quality of life (QOL) [28] and prognosis [20, 21] of CHF patients. Despite the fact patients with advanced CHF accounted for the majority of patients in the present study, left ventricular dimension reduced and systolic function improved after ASV therapy; namely, left ventricular reverse remodeling occurred. Furthermore, comparisons between the pre- and post-ASV values and the time-series analysis of the data obtained revealed decreases in blood pressure and reductions in CTR and BNP [18, 23]. These results raise an expectation that ASV may improve the prognosis of CHF patients.

The improvement rate of LVEF concerning patients with impaired systolic function at week 26 of treatment in the present study was approximately 5 % and was equivalent to the improvement rates of LVEF obtained in 3-month continuous positive airway pressure [29], 16-month ASV therapy [30], 6-month pharmacotherapy [31], and meta-analysis of CRT [32] (5, 7, 7, and 5.9 %, respectively). In consideration of the fact that all patients had already undergone sufficient CHF therapy at the onset of ASV therapy, this result indicates the sufficient therapeutic relevance of ASV therapy. The factors associated with LVEF improvement in the present study were the low onset value of LVEF, advanced age, and absence of CKD. SDB severity was not related to LVEF improvement. The findings described above suggest that the effects of ASV therapy on patients with CHF complicated by SDB were not exerted through the treatment of SDB, but were based on the improvement in patients’ hemodynamics. Namely, the direct effects on hemodynamics are probably translated into the beneficial effects of NPPV therapy, e.g., inhibition of sympathetic nerve activity [29, 33] and improvement in QOL [24, 31, 34], which have been reported in previous clinical studies in the relevant patients.

The present study has several limitations. First, sample size is relatively small. The device was launched in December 2007 in Japan, and most of the patients initiated to undergo ASV therapy using the device shortly thereafter. Therefore, the number of patients at 16 medical institutions was as relatively small as 115. We expect findings in the present study to be verified in a larger scale clinical study. Second, no definitive conclusions can be drawn because the present study is not a randomized controlled study, nor any precise diagnosis of SDB could be made because overnight polysomnography was not performed in real-world clinical settings. Nevertheless, improvements in NYHA class are highly likely to be attributable to ASV therapy. Third, information bias cannot be ruled out because the present study is retrospective in design. A prospective randomized placebo-controlled study is required to solve these regards.

In conclusion, real-world practice in Japan was evidenced where ASV therapy is applied to patients with relatively severe CHF, regardless of the presence or absence of SDB. The present study suggests the following: (1) CHF patients present long-term ASV continuity that is affected not by SDB severity but by CHF severity; and (2) ASV therapy improves symptoms, left ventricular contractility, and remodeling in a not SDB but CHF severity-dependent manner. Therefore, ASV therapy is expected to become a novel and promising non-pharmacotherapy for relevant patients. A randomized controlled study to verify these effects is warranted.

## Appendix: The SAVIOR-R Investigators

The SAVIOR-R Investigators Gunma Prefectural Cardiovascular Center: H. Adachi; National Cerebral and Cardiovascular Center: H. Yokoyama; Saitama Medical Center, Jichi Medical University: S.I. Momomura, C. Suga, Y. Sugawara, N. Ikeda, A. Obara; Hiroshima University: Y. Kihara, N. Oda; Tohoku University: H. Shimokawa, Y. Fukumoto; Hyogo College of Medicine: T. Masuyama, M. Kawabata; Toyama University: H. Inoue, S. Joho; Nagoya University: T. Murohara, A. Hirashiki; Nippon Medical School Chiba Hokusoh Hospital: Y. Seino, T. Inami; Osaka National Hospital: Y. Yasumura, M. Koide; Keio University: T. Yoshikawa, S. Mogi; Mie University: M. Ito, K. Dohi; Saiseikai-Futsukaichi Hospital: S. Ando; Tokushima University: M. Sata, Y. Taketani; Imizu Municipal Hospital: H. Asanoi, H. Ueno; Kumamoto University: H. Ogawa, M. Yamamuro.
